# Dietary *Phaffia rhodozyma*-Synthesized 3S, 3′S-Astaxanthin Promotes Body Coloration and Muscle Quality in Pacific White Shrimp *Litopenaeus vannamei*

**DOI:** 10.1155/anu/9993234

**Published:** 2025-03-19

**Authors:** Beili Zhang, Chunyang Zhang, Jianing Xu, Wenyi Wang, Chengguo Zhang, Juan Tian, Chaoqun Li, Qinyuan Ma

**Affiliations:** ^1^School of Life Sciences and Medicine, Shandong University of Technology, Zibo 255000, China; ^2^Joint Institute of Synthetic Biology and Engineering Biotechnology, Shandong University of Technology and Jincheng Pharma, Zibo 255000, China; ^3^Yangtze River Fisheries Research Institute, Chinese Academy of Fishery Sciences, Wuhan 430223, China

**Keywords:** 3S, 3′S-astaxanthin, body coloration, *Litopenaeus vannamei*, muscle quality, nutritional composition, texture

## Abstract

The focus of people on the yield of aquatic products has gradually shifted to superior quality. Astaxanthin is well-known for its superior antioxidant capacity, while research on its regulatory effect on muscle quality is limited. This study aims to investigate whether dietary *Phaffia rhodozyma*-synthesized 3S, 3′S-astaxanthin (L-AST) could promote the body coloration and muscle quality of *Litopenaeus vannamei*. Experimental diets with L-AST levels of 0, 30, 60, and 90 mg/kg were fed to *L. vannamei* (IBW: 2.72 ± 0.03 g) for 8 weeks. The results revealed that the shrimp fed L-AST diets presented better body coloration with decreased L^*∗*^ values whereas increased a^∗^ values and possessed higher levels of muscle astaxanthin. Dietary supplementation with 60 mg/kg L-AST significantly enhanced muscle texture (hardness, chewiness, resilience, and gumminess), which could be attributed to an increase in alkaline-insoluble collagen content and a decrease in myofiber diameter. Interestingly, dietary supplementation with 60 or 90 mg/kg L-AST markedly increased the content of glycine (a sweet amino acid) and total flavor amino acid (glutamic acid, aspartate, alanine, glycine). Further study showed that dietary supplementation with 60 or 90 mg/kg L-AST significantly improved free fatty acid profile by increasing contents of some monounsaturated fatty acids (C17:1n7, C18:1n9t and C24:1n9) and polyunsaturated fatty acids (C20:02 and C22:6n3). Taken together, dietary *P. rhodozyma*-synthesized L-AST considerably promoted muscle quality in *L. vannamei* by promoting histological and texture properties, elevating alkaline-insoluble collagen content while improving the profile of free amino acids and fatty acids.

## 1. Introduction

Shrimps help meet the needs of a growing population for nutritious food. Shrimp meat is a source of high-quality protein with a decreased fat level and a key source of essential polyunsaturated fatty acids and amino acids for humans [1]. Due to an improvement in living standards and the upgradation of consumption concepts, the focus of people on the yield of aquatic products has shifted to superior quality. Muscle represents the major edible part in shrimp, whose quality is the major factor affecting quality classification [2]. Considering these facts, green and efficient regulation strategies need to be developed to improve the muscle quality of shrimp.

Muscle quality can be evaluated based on several aspects, including sensory quality, nutritive value, texture properties, flavor, and hygiene [3, 4]. For example, regarding texture properties, people generally prefer aquatic products with high hardness, resilience, and gumminess, and such superior texture properties depend mainly on the nutritional composition and histological properties of the muscle [5–7]. Along with the farmed species, environmental conditions, feeding management, and other factors, dietary nutrients also strongly affect muscle quality. Cheng et al. [8] reported that dietary creatine can promote muscle quality in *L. vannamei* by increasing hardness, chewiness, and the contents of sweet flavor and muscular protein. Shi et al. [9] reported that dietary supplementation of hydroxyproline in low-fish meal diets can improve the collagen content, myofiber characteristics, and texture, thus improving the flesh quality of juvenile *L. vannamei*.

Astaxanthin (3,3′-dihydroxy-4,4-diketo-*β*,*β*′-carotene) accounts for the xanthophyll carotenoid with three stereoisomers (3S, 3′S, 3S, 3′R, and 3R, 3′R), which cannot be produced de novo through aquatic animals [10]. Astaxanthin is renowned for its superior antioxidant capacity and is also widely used as a pigment for several farmed fish, such as salmonid and rainbow trout [11, 12]. In shrimp, astaxanthin has multiple functions, such as improving body color and enhancing immunity, antioxidation, and antistress abilities [13]; however, research on its regulatory effect on muscle quality is still limited. Moreover, compared to bioextracted and chemically synthesized astaxanthin, little is known about the physiological functions of *P. rhodozyma*-synthesized 3S, 3′S-astaxanthin (L-AST) in shrimp. Thus, we selected the Pacific white shrimp *Litopenaeus vannamei* to be the experimental animal and L-AST (synthesized using *P. rhodozyma* as the chassis cell) as a nutritional regulator. We evaluated whether dietary *P. rhodozyma*-synthesized L-AST promotes the muscle quality of *L. vannamei* in terms of coloration, astaxanthin content, collagen content, histomorphology, texture prospects, and free amino acid and fatty acid profiles.

## 2. Materials and Methods

### 2.1. Experimental Design

Every experiment gained approval from Animal Welfare Ethics Committee of Shandong University of Technology. *P. rhodozyma*-synthesized L-AST was obtained from Shandong Jincheng Pharmaceutical Group Co., Ltd. Four L-AST diets at 0, 30, 60, and 90 mg/kg were prepared by adding L-AST-rich *P. rhodozyma* to the basal feed (defined as PRLA0, PRLA30, PRLA60, and PRLA90). The basal feed formula and preparation process were identical to those in our previous study [14] and are shown in Table 1. The individuals of Pacific white shrimp *L. vannamei* (initial body weight: 2.72 ± 0.03 g) were divided into four groups based on the L-AST supplementation level, with three 120-L tanks per dietary group and 40 shrimp per tank. The different diets were given to 4% of the body weight four times a day (7:00 a.m., noon, 4:00 p.m., and 8:00 p.m.) for an 8-week period. During the rearing period, we changed about 40% of the water in each tank every day and kept environmental conditions below 27–29°C water temperature, pH 7.0–8.0, ammonia-N <0.002 mg/L, and 6–7 mg/L dissolved oxygen.

### 2.2. Sampling Procedures

After feeding test, shrimp from each tank experienced a 24-h starving period, weighed, and counted after anesthesia. Three shrimp per tank were boiled for color analysis. We harvested muscle samples from 15 shrimp/tank and preserved them under −80°C to analyze their nutrient composition and contents of astaxanthin, collagen, free amino acids and free fatty acids. We obtained muscle samples in three shrimp per tank and fixed in 4% paraformaldehyde for the preparation and analysis of paraffin sections. Another three shrimps per tank were cooked, the shell was peeled off, and the muscle samples from the second abdominal segments were used immediately for texture analysis.

### 2.3. Body Color

For analyzing the body color, the second abdominal segments of the shrimp (cooked for 3 min in 100 mL of water at 100°C) were determined using a WSC-S colorimeter (Physical Optics Instrument Factory of Shanghai Precision Scientific Instrument Co., Ltd., Shanghai, China) calibrated against a white reference plate [15]. The values of lightness (L^*∗*^), yellowness (b^∗^) and redness (a^∗^) were recorded to evaluate body coloration status.

### 2.4. Muscle Astaxanthin Content and General Nutritional Composition Analyses

Astaxanthin content was detected according to Parisenti et al. [16] after modification. The muscle samples (8 g) were ground, adequately homogenized, and extracted three times (2 min each) in 30 mL of precooled acetone. Then, it was centrifuged at 5000 *r*/min and 4°C for a 5-min duration, after which supernatant was harvested. The mixed supernatant was added to a separation funnel, and hexane (25 mL) and 0.5% NaCl solution (100 mL) were added. The mixture was evenly shaken by hand and extracted for 20 min. Then, the hexane extract was filtered and dehydrated using anhydrous sodium sulfate prior to transfer to the 25-mL volumetric flask with a constant volume. Thereafter, astaxanthin content in hexane extracts was detected via high-performance liquid chromatography (HPLC). Besides, a freeze-drying approach was used to measure moisture content. A Soxhlet extraction approach was used for measuring the crude lipid level. Kjeldahl approach was used to measure the crude protein level. After combustion in the muffle furnace at 550°C, we measured the crude ash level.

### 2.5. Collagen Content Analyses

Collagen content analysis was conducted according to the descriptions of previous studies [17, 18]. Briefly, 1 g of muscle sample was homogenized to nine times the volume of precooled distilled water. Then, precooled NaOH solution (10 mL, 0.2 M) was added and shaken for a 4-h duration under 4°C, prior to 30 min of centrifugation at 10,000 ×*g* to obtain supernatant and precipitate (alkaline-soluble hydroxyproline represents soluble collagen, whereas alkaline-insoluble hydroxyproline represents insoluble collagen). The precipitate was transferred into ampoules, followed by the addition of 3 mL HCl solution (6 M). Later, this resultant mixture was sealed with an alcohol blowtorch, hydrolyzed for a 20-h duration under 110°C, and cooled till ambient temperature; the volume was fixed to 10 mL. Next, 1 mL of the supernatant was incubated with 2 mL of buffered chloramine T reagent under ambient temperature for 20 min. Thereafter, we introduced 2 mL perchloric acid and incubated this mixture for a 5-min duration under ambient temperature. When 2 mL 4-dimethylaminobenzaldehyde solution was added, the mixed sample was heated under 60°C for a 20-min duration and cooled till ambient temperature. Absorbance values were detected at 560 nm, and the final level was measured based on hydroxyproline standard curve.

### 2.6. Muscle Histological Analysis

Muscle histological analysis was conducted following a previous study by Cheng et al. [8]. After 24 h of fixation with 4% paraformaldehyde solution, muscle tissue samples from the second abdominal segment (0.5 × 0.5 × 0.5 cm) were dehydrated with ethanol and xylene, followed by paraffin embedding and HE staining. At last, the digital microscope (DA1-180 M) was adopted for observing those stained slices using the ScopeImage 9.0 software.

### 2.7. Texture Analysis

Cooked muscle samples from the second abdominal segment were used for texture profile analysis (TPA) [15]. We measured texture parameters, such as chewiness, hardness, resilience, gumminess, springiness, and cohesiveness, with the TVT-300XP texture analyzer (Bothong Ruihua Scientific Instrument Beijing Co., Ltd., Beijing, China). We adopted the P-cy5s cylindrical probe in two tests, with speed before and following the test being set at 2 mm/s, test speed at 1 mm/s, interval at 5 s, data acquisition rate at 200 pps, and a compression ratio of 60%.

### 2.8. Free Amino Acid Measurement

We detected free amino acid level as described by Xu et al. [19] after minor modification, the muscle samples were removed from the −80°C freezer and freeze-thawed at 4°C. After completely melting, the muscle samples were weighed and put into the 5-mL centrifuge tube. Then, 10% sulfosalicylic acid at three-fold volume was introduced, fully homogenized, and left undisturbed for 5 min. Following 15 min of centrifugation under 4°C (13,000 ×*g*), supernatants were collected and passed through the 0.22-*μ*m filter membrane. Finally, free amino acid content was measured through an automated amino acid detector (HITICHI L-8900, Tokyo, Japan).

### 2.9. Free Fatty Acid Measurement

We determined the levels of muscle-free fatty acids by gas chromatography (Agilent 7890 A) following the previous study by Liu et al. [20] with some modifications. In detail, 0.4 g freeze-dried muscle samples were thoroughly blended using 4 mL isooctane and placed on a shaking table under 25°C for overnight extraction. Next, 2% sodium hydroxide methanol (8 mL) was introduced into this extraction mixture prior to 40 min of gentle boiling. After adding 15% boron trifluoride methanol (7 mL), this resultant mixed sample was gently boiled for 20 min. Following this, we introduced n-heptane (20 mL) and gently boiled this mixed sample, for 1 min. Following the addition of a saturated sodium chloride solution and standing of this mixed sample, the upper n-heptane extraction mixture (5 mL) was obtained and added into anhydrous sodium sulfate (5 g, 1 min of shaking and 5 min of standing). Finally, we transferred the upper layer of solution to the sample bottle for determination. Fatty acid content was calculated based on peak area ratio of different fatty acids to internal standard (C11:0).

### 2.10. Statistical Analysis

All data were evaluated for homogeneity and normality. Among-group differences were analyzed by one-way ANOVA plus Tukey's test using IBM SPSS 22.0 data analysis software. *p* < 0.05 stood for statistical significance. The final data were represented by the mean ± standard error.

## 3. Results

### 3.1. Body Color

**Figure 1:**
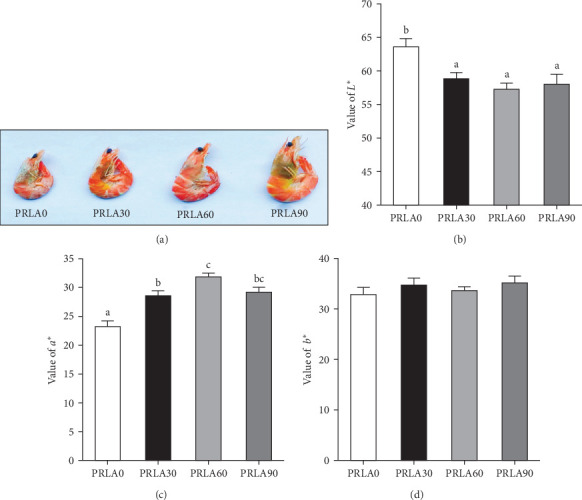
Comparison of body coloration of *Litopenaeus vannamei* fed different diets. (A) Visual color observation; (B) value of L^*∗*^; (C) value of a^*∗*^; (D) value of b^*∗*^. L^*∗*^, lightness; a^*∗*^, redness; b^*∗*^, yellowness. Values are displayed as means ± standard error (*n* = 3). Different letters (a, b, c) above the bars represent statistical differences based on Tukey's test (*p* < 0.05, one-way ANOVA).

Visual color observation presented that dietary L-AST markedly enhanced the body pigmentation of shrimp (Figure 1A). Compared to those in the PRLA0 group, the shrimp in the three L-AST-fed groups presented decreased L^*∗*^ values whereas increased a^∗^ values (*p* < 0.05**)** (Figure 1B,C). b^∗^ values were not significantly different across the dietary groups (*p* > 0.05) (Figure 1D).

### 3.2. Muscle Astaxanthin Content and General Nutritional Composition

**Figure 2:**
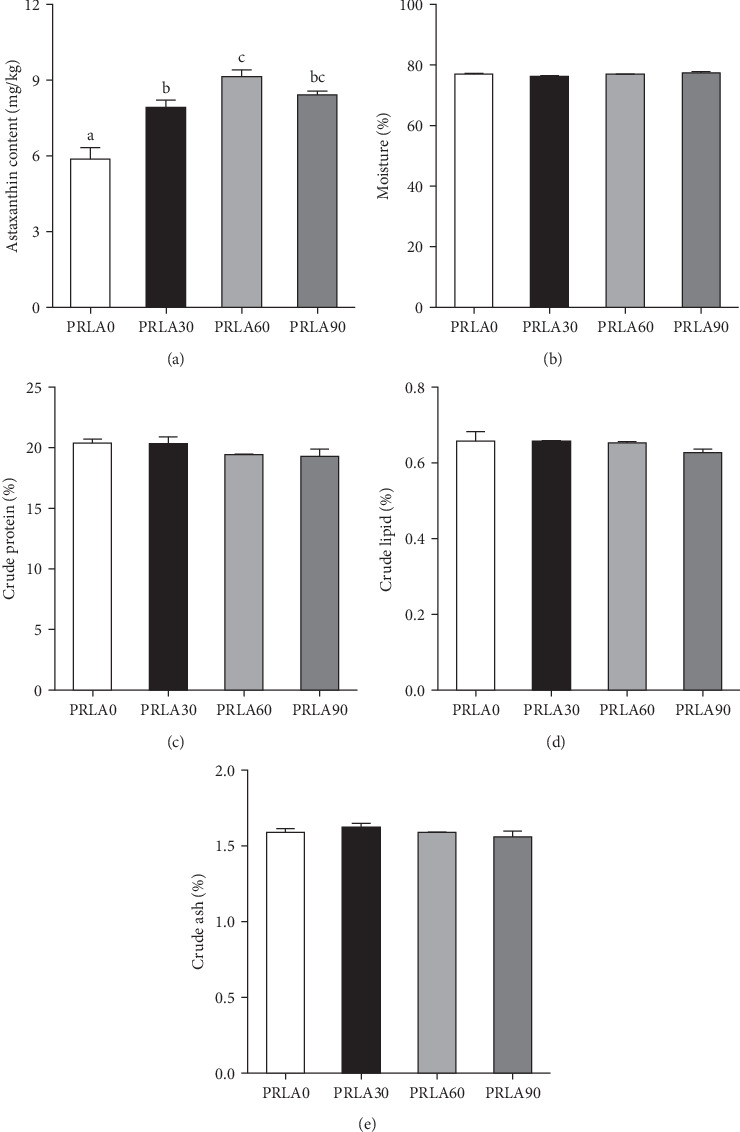
Astaxanthin content and general nutritional composition in the muscle of *Litopenaeus vannamei* fed different diets. (A) Astaxanthin content in the muscle of shrimp. (B–E) General nutritional composition including moisture (B), crude protein (C), crude lipid (D), and crude ash (E) content in the muscle of shrimp. Values are displayed as means ± standard error (*n* = 3). Different letters (a, b, c) above the bars represent statistical differences based on Tukey's test (*p* < 0.05, one-way ANOVA).

The muscle astaxanthin content was significantly higher in all three L-AST-fed groups than PRLA0 group (*p* < 0.05) (Figure 2A). However, general nutritional parameters, including moisture, crude protein, crude lipid, as well as crude ash levels, were not significantly different across the four dietary groups (*p* > 0.05) (Figures 2b-2e).

### 3.3. Collagen Content

**Figure 3:**
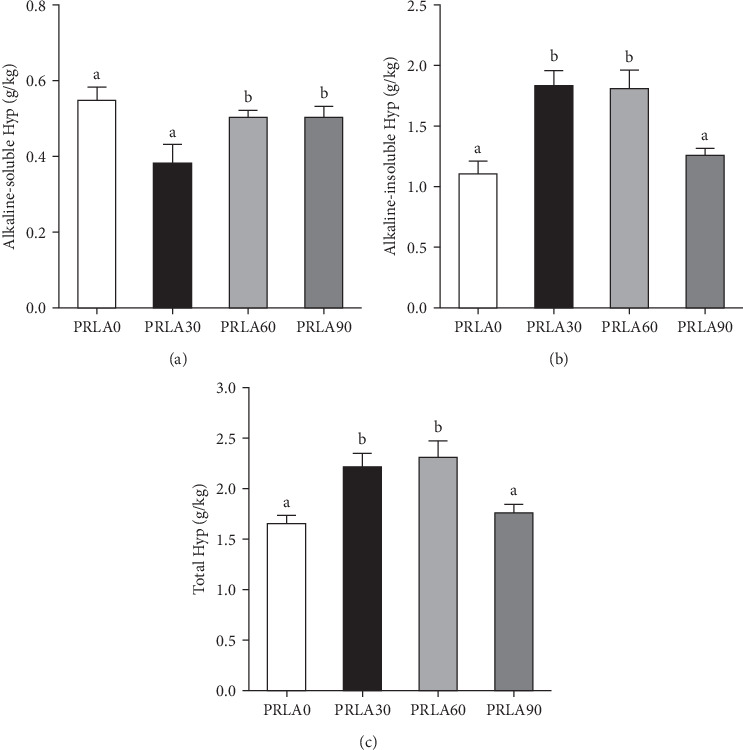
Collagen content in the muscle of *Litopenaeus vannamei* fed different diets. (A) Alkaline-soluble collagen content; (B) alkaline-insoluble collagen content; (C) total collagen content. Values are displayed as means ± standard error (*n* = 3). Different letters (a, b) above the bars represent statistical differences based on Tukey's test (*p* < 0.05, one-way ANOVA).

In comparison with PRLA0 group, alkaline-soluble collagen levels of PRLA60 and PRLA90 groups did not show any significant difference (*p* > 0.05), whereas that of PRLA30 group remarkably decreased (*p* < 0.05) (Figure 3A). Furthermore, alkaline-insoluble collagen and total collagen levels of PRLA30 and PRLA60 groups remarkably increased (*p* < 0.05), whereas PRLA90 group did not show any significant difference (*p* > 0.05) (Figure 3B,C).

### 3.4. Muscle Histomorphology

**Figure 4:**
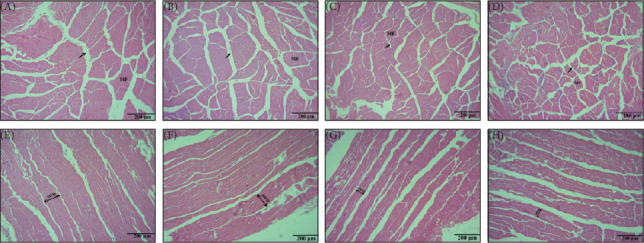
The muscle morphology of *Litopenaeus vannamei* fed different diets. (A–D) Representative cross sections in the PRLA0 (A), PRLA30 (B), PRLA60 (C), and PRLA90 (D) group; (E–H) Representative longitudinal sections in the PRLA0 (A), PRLA30 (B), PRLA60 (C), and PRLA90 (D) group.

The micromorphological changes in the muscle of the different dietary groups can be observed in Figure 4. Relative to PRLA0 group, L-AST-fed groups, especially the PRLA 60 and PRLA 90 groups, presented significantly smaller myofiber diameters and gaps (*p* < 0.05), indicating that the shrimp in the L-AST-fed groups presented greater muscle compactness.

### 3.5. Texture Characteristics of Muscle

**Figure 5:**
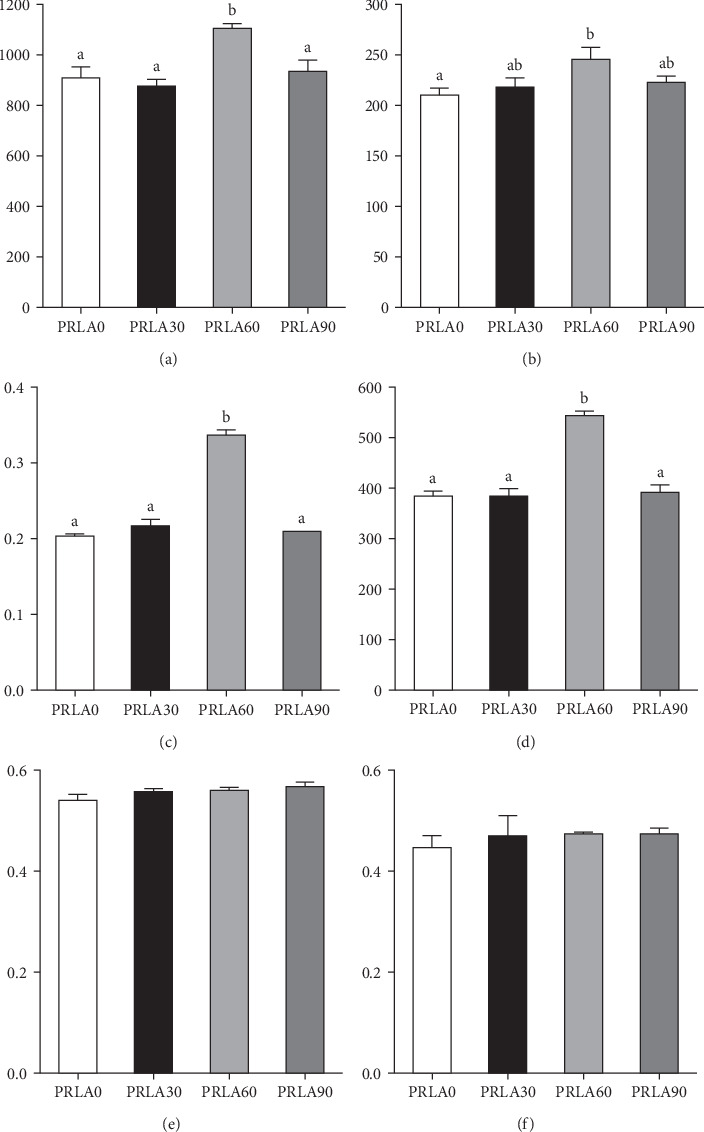
The muscle texture of *Litopenaeus vannamei* fed different diets. (A) Hardness; (B) Chewiness; (C) Resilience; (D) Gumminess; (E) Springiness; (F) Cohesiveness. Values are displayed as means ± standard error (*n* = 3). Different letters (a, b) above the bars represent statistical differences based on Tukey's test (*p* < 0.05, one-way ANOVA).

As shown in Figure 5, muscle springiness or cohesiveness was not significantly different across diverse dietary groups (*p* > 0.05). Nonetheless, the PRLA60 group presented significantly greater muscle hardness, chewiness, resilience, and gumminess than the PRLA0 group (*p* < 0.05).

### 3.6. Muscle-Free Amino Acid Level

From Table 2, the muscle total free amino acid level markedly increased PRLA90 group relative to the PRLA0 group (*p* < 0.05). Essential amino acid level was not significantly different across the dietary groups (*p* > 0.05). But glycine (the sweet amino acid) level significantly increased in the three L-AST-fed groups relative to PRLA0 group (*p* < 0.05). Additionally, total flavor amino acid (aspartate, glutamic acid, glycine, and alanine) level in PRLA60 and PRLA90 groups increased relative to PRLA0 group (*p* < 0.05). Nonessential amino acids, serine, tyrosine, and histidine levels showed no significant differences among dietary groups (*p* > 0.05), whereas contents of arginine, proline, and total nonessential amino acids remarkably increased in PRLA90 relative to PRLA0 groups (*p* < 0.05).

### 3.7. Muscle-Free Fatty Acid Level

Muscle-free fatty acid profile of the shrimps is shown in Table 3. The C22:0 content in the PRLA90 group remarkably increased relative to PRLA0 group (*p* < 0.05), and differences in the contents of other saturated fatty acids were not significant among dietary groups. Relative to that in PRLA0 group, C17:1n7 level apparently increased in three L-AST-fed groups (*p* < 0.05), C18:1n9t level apparently elevated in PRLA60 group (*p* < 0.05), while C24:1n9 level dramatically increased in PRLA90 group (*p* < 0.05). For polyunsaturated fatty acids, the contents of C20:02 in the PRLA60 group and C22:6n3 in all three L-AST-fed groups markedly elevated relative to PRLA0 group (*p* < 0.05).

## 4. Discussion

Consumers give importance to the body color of shrimp, as it can visually reflect the degree of freshness, nutritive quality, and health status of the shrimp [16]. Thus, body color is an important criterion for the commercial value of shrimp, and consumers usually prefer shrimps with darker colors and greater redness (a^∗^) [15]. In our study, the shrimp in the L-AST-fed groups presented lower lightness (L^*∗*^) and higher a^∗^, indicating that dietary L-AST markedly improved the body coloration of *L. vannamei*. Previous studies have also indicated that dietary astaxanthin can improve body coloration of various shrimp species, such as Pacific white shrimp (*L. vannamei*), black tiger prawn (*Penaeus monodon*), kuruma shrimp (*Marsupenaeus japonicus*), and peppermint shrimp (*Lysmata wurdemanni*) [21–24]. Astaxanthin is the most important carotenoid that determines the body color of shrimp. Along with the results of body coloration, all L-AST-fed groups presented higher muscle astaxanthin contents than the control group. Similar findings were obtained by Honda et al. [25] and Zhang et al. [26].

Collagen, a major connective tissue component, has an important effect on maintaining the mechanical strength, integrity, and rheological properties of muscles, and its content is often expressed as the content of hydroxyproline [5, 8]. Although the collagen content in prawn muscle is very low, it strongly affects muscle quality. In this study, the general nutritional parameters, including moisture, crude protein, crude lipid, and crude ash levels, were not significantly different across the dietary groups; however, 30 and 60 mg/kg L-AST significantly increased the alkaline-insoluble collagen and total collagen contents in muscle. Research concerning the functions of dietary astaxanthin in muscle collagen content in prawns is lacking, but previous studies have shown that the content, degree of molecular aggregation, and stability of collagen, especially alkaline-insoluble collagen, are positively correlated with muscle texture characteristics (including hardness and chewability) in aquatic animals. Moreno et al. [27] reported that muscle firmness in Atlantic salmon (*Salmo salar* L.) was positively correlated with stable collagen with more triple helix structures and a greater degree of glycosylation. As discovered by Li et al. [15], the reduced alkaline-insoluble collagen level within *Chlorella*-fed *L. vannamei* contributed to decreased muscle hardness and shear force.

The histological characteristics, including myofiber diameter, spacing, and density, are also key factors affecting muscle quality, such as color, texture, and flavor [4]. In livestock, muscles with smaller myofiber diameters have greater water-holding capacity, lower shear force, and greater tenderness [28]. However, in aquatic animals, myofibers with smaller diameters and greater densities can usually improve the tightness, hardness, and even coloration of muscle [8, 29]. For example, a study revealed that a high myofiber density was associated with a firm texture and better coloration in Atlantic salmon (*S. salar* L.) muscle [30]. In this study, L-AST-fed groups, especially the PRLA60 and PRLA90 groups, presented significantly smaller myofiber diameters and gaps, indicating that the shrimp in the L-AST-fed groups might have greater muscle compactness. The aforementioned results concerning collagen and histological characteristics motivated us to investigate whether dietary L-AST can lead to changes in muscle texture properties.

The texture properties, including hardness, chewiness, resilience, gumminess, etc., are the most intuitive indicators reflecting the quality of shrimp muscle. Consumers often exhibit significantly different preferences for the muscle texture of livestock and aquatic animals. In livestock, muscle quality is positively correlated with muscle tenderness. However, in aquatic animals, consumers prefer muscle textures with high hardness, resilience, and compactness [6, 31]. According to our results, dietary supplementation with 60 mg/kg L-AST dramatically increased muscle hardness, chewiness, resilience, and gumminess. In the recent research regarding rainbow trout, dietary supplementation of 75–125 mg/kg astaxanthin obtained from wall-broken *H. pluvialis* improved the fillet texture [32]. Alkaline insoluble collagen molecules have a high degree of cross-linking, and an increase in their content helps form an elastic network and enhances the tensile strength of muscle [33]. A study on Atlantic halibut (*Hippoglossus hippoglossus* L.) revealed that the degree of cross-linking and content of collagen had a significantly greater effect on hardness than the myofiber density [34]. Therefore, in our study, the increase in the content of alkaline insoluble collagen probably exerted an important effect on enhancing muscle texture.

Amino acid content and types are important nutritional indices for evaluating muscle quality. Amino acid pyrolysis and the Maillard reaction are crucial chemical reactions that form different flavors of meat [35, 36]. The sweetness of fresh shrimp is mainly determined by muscle-free amino acid composition and content, particularly flavor amino acid contents, like aspartate, glutamic acid, alanine, and glycine [8, 37]. In this study, we analyzed how dietary L-AST affected the muscle amino acid profile of *L. vannamei*, and the results revealed that the content of glycine in the L-AST-fed groups significantly increased, and total flavor amino acid (aspartate, glutamic acid, alanine, and glycine) levels markedly elevated when L-AST concentration was 60 mg/kg. No previous study has analyzed how dietary biosynthesized astaxanthin affected amino acid profile in prawn muscle. The transcriptome and metabolome analysis in *Exopalaemon carinicauda* suggested that amino acid metabolic pathways were upregulated in the muscle after feeding astaxanthin [10]. These findings suggested that L-AST might alter the amino acid profile in muscle by affecting amino acid metabolism; however, further research is needed to confirm this speculation.

Fatty acid profile represents another important index reflecting muscle quality in aquatic animals. Volatile compounds derived from unsaturated fatty acids under the action of lipoxygenase can result in a fragrant aroma. Lipid peroxidation damages the integrity of biofilms, and the resulting aldehyde and alcohol complexes can cause muscle decay and emit a strong odor [38]. For example, the muscle n-3 fatty acids within turbot (*Psetta maxima*) give the fish an aromatic smell [39], whereas n-6 fatty acids give tench muscle (*Tinca tinca* L.) an unpleasant odor [40]. According to our results, saturated fatty acid levels (C22:0), three monounsaturated fatty acids (C17:1n7, C18:1n9, and C24:1n9), and two polyunsaturated fatty acids (C20:02 and C22:6n3) significantly increased after proper L-AST feeding. Similarly, a study on black tiger prawn (*P. monodon*) revealed that synthetic astaxanthin performed better than natural astaxanthins with regard to n-3 polyunsaturated fatty acid deposition within muscle [41]. Such an improved fatty acid profile after L-AST supplementation might also contribute to favorable changes in muscle texture.

## 5. Conclusion

To summarize, this study performed the first assessment on the role of *Phaffia rhodozyma*-synthesized L-AST in the muscle quality regulation in aquatic animal. The findings of this study showed that dietary L-AST strongly promoted body coloration and muscle astaxanthin content in *L. vannamei*. Dietary L-AST also considerably promoted *L. vannamei* muscle quality via enhancing histological and texture properties, increasing alkaline-insoluble collagen content, and improving the profile of free amino acids and fatty acids. Our results help further understand physiological function of biosynthetic L-AST and provide solution for the improvement of shrimp muscle quality.

## Figures and Tables

**Table 1:** Basal feed formula and composition (% dry matter).

Ingredients	Basal feed
Fishmeal	20.00
Soybean meal	25.00
Peanut meal	15.00
Wheat flour	21.49
Wheat gluten meal	3.51
Squid paste	3.00
Cellulose	3.75
Fish oil	1.80
Phospholipid	1.00
Monocalcium phosphate	1.50
Choline chloride	0.50
Calcium propionic acid	0.10
Ethoxyquin	0.05
Sodium alginate	0.30
Mineral premix^a^	1.50
Vitamin premix^b^	1.50
Proximate composition (% dry matter)	
Crude protein	43.03
Crude lipid	6.49
Crude ash	9.38

^a^Mineral premix contained (g/kg mixture): KCl, 90.00; KI, 0.04; NaCl, 40.00; CuSO_4_ · 5H_2_O, 3.00; ZnSO_4_ · 7H_2_O, 4.00; CoSO_4_·7H_2_O, 0.02; FeSO_4_ · 7H_2_O, 20.00; MnSO_4_ · H_2_O, 3.00; MgSO_4_ · 7H_2_O, 124.00; CaHPO_4_·2H_2_O, 500.94; CaCO_3_, 215.00.

^b^Vitamin premix contained (g/kg mixture): retinyl acetate, 2.50; VD3, 6.25; all-racatocopheryl acetate, 75.00; VK, 2.50; VB1, 0.25; VB2, 1.00; D-calcium pantothenate; 5.00; VB6, 0.75; VB12, 2.50; niacin, 2.50; folic acid 0.25; biotin 2.50; meso-inositol, 379.00.

**Table 2:** Muscle-free amino acid levels in *Litopenaeus vannamei* (mg/100g).

Amino acids	Treatments			
	PRLA0	PRLA30	PRLA60	PRLA90
Thr	17.66 ± 1.75	20.39 ± 3.16	20.60 ± 2.49	20.19 ± 0.46
Val	33.04 ± 1.48	26.50 ± 2.66	28.46 ± 2.19	32.70 ± 1.12
Leu	26.12 ± 2.48	26.15 ± 3.18	27.75 ± 2.67	30.28 ± 0.14
Ile	17.90 ± 1.41	16.97 ± 1.73	16.95 ± 1.64	18.13 ± 0.38
Met	3.66 ± 0.03	3.72 ± 0.15	3.37 ± 0.54	3.54 ± 0.30
Phe	12.64 ± 0.96	13.60 ± 0.88	11.95 ± 1.40	13.17 ± 0.02
Lys	20.21 ± 0.55	25.11 ± 4.12	23.21 ± 2.70	24.93 ± 0.50
ΣEAA	131.22 ± 7.28	132.45 ± 15.72	132.29 ± 12.97	142.95 ± 1.07
Asp	4.55 ± 0.37^ab^	5.03 ± 0.29^b^	3.75 ± 0.22^a^	5.10 ± 0.28^b^
Glu	37.78 ± 0.54^ab^	38.13 ± 1.59^b^	33.88 ± 1.59^a^	39.75 ± 0.91^b^
Gly	150.93 ± 1.04^a^	164.93 ± 5.00^b^	164.29 ± 2.72^b^	166.45 ± 4.70^b^
Ala	169.97 ± 2.77	180.67 ± 18.02	165.63 ± 6.90	177.00 ± 8.21
ΣFAA	363.23 ± 1.46^a^	388.76 ± 2.67^b^	367.54 ± 5.86^a^	388.29 ± 5.22^b^
Ser	2.25 ± 0.10	2.23 ± 0.11	2.10 ± 0.13	2.25 ± 0.08
Tyr	16.47 ± 0.28	17.68 ± 1.44	18.71 ± 0.10	19.11 ± 0.45
His	25.11 ± 2.71	24.82 ± 0.96	22.16 ± 1.60	25.66 ± 1.07
Arg	471.09 ± 3.78^a^	464.49 ± 5.24^a^	464.26 ± 17.78^a^	510.99 ± 1.43^b^
Pro	285.50 ± 17.25^a^	309.70 ± 22.92^ab^	337.00 ± 5.83^ab^	355.92 ± 19.69^b^
ΣAA	1294.87 ± 27.79^a^	1340.14 ± 10.96^a^	1344.06 ± 26.01^a^	1445.16 ± 24.75^b^

*Note:* Data are represented by means ± standard error (*n* = 3). Means in one column with diverse superscripts show significant differences (*p* < 0.05).

Abbreviations: ΣAA, total amino acids; ΣEAA, total essential amino acids; ΣFAA, total flavor amino acids.

**Table 3:** Muscle-free fatty acid levels in *Litopenaeus vannamei* (% total fatty acids).

Fatty acids	Treatments			
	PRLA0	PRLA30	PRLA60	PRLA90
C14:0	0.51 ± 0.03	0.49 ± 0.02	0.46 ± 0.02	0.51 ± 0.05
C15:0	0.53 ± 0.01	0.49 ± 0.01	0.45 ± 0.01	0.45 ± 0.05
C16:0	20.41 ± 0.08	20.56 ± 0.08	20.34 ± 0.05	20.42 ± 0.14
C17:0	1.11 ± 0.06	1.07 ± 0.02	1.02 ± 0.02	1.01 ± 0.03
C18:0	11.09 ± 0.15	11.2 ± 0.10	11.34 ± 0.07	11.03 ± 0.15
C20:0	0.69 ± 0.03	0.63 ± 0.05	0.66 ± 0.05	0.73 ± 0.05
C22:0	0.67 ± 0.01	0.68 ± 0.06	0.66 ± 0.03	0.84 ± 0.04^b^
ΣSFA	35.02 ± 0.12	35.12 ± 0.15	34.93 ± 0.13	34.99 ± 0.21
C16:1n7	1.28 ± 0.08	1.36 ± 0.04	1.21 ± 0.01	1.21 ± 0.05
C17:1n7	0.18 ± 0.01^a^	0.20 ± 0.01^b^	0.20 ± 0.00^b^	0.22 ± 0.01^c^
C18:1n9t	0.19 ± 0.01^a^	0.21 ± 0.01^a^	0.25 ± 0.00^b^	0.21 ± 0.01^a^
C18:1n9c	12.82 ± 0.12	12.34 ± 0.10	12.44 ± 0.11	12.42 ± 0.28
C20:01	1.06 ± 0.04	1.07 ± 0.03	1.08 ± 0.05	1.12 ± 0.02
C24:1n9	0.37 ± 0.03^a^	0.35 ± 0.03^a^	0.38 ± 0.03^a^	0.51 ± 0.02^b^
ΣMUFA	15.90 ± 0.17	15.52 ± 0.07	15.56 ± 0.16	15.70 ± 0.19
C18:2n6c	17.72 ± 0.21	17.36 ± 0.19	17.21 ± 0.16	17.18 ± 0.25
C18:3n3	1.27 ± 0.04	1.20 ± 0.07	1.22 ± 0.01	1.24 ± 0.03
C20:02	1.28 ± 0.05^a^	1.29 ± 0.05^a^	1.50 ± 0.01^b^	1.36 ± 0.06^ab^
C20:4n6	2.02 ± 0.02	1.96 ± 0.03	2.08 ± 0.08	2.13 ± 0.15
C20:5n3	12.95 ± 0.12	13.40 ± 0.03	13.31 ± 0.12	13.32 ± 0.24
C22:6n3	13.77 ± 0.02^a^	14.15 ± 0.02^b^	14.18 ± 0.03^b^	14.09 ± 0.06^b^
ΣHUFA	49.07 ± 0.09	49.36 ± 0.14	49.51 ± 0.29	49.31 ± 0.31

*Note:* Data are represented by means ± standard error (*n* = 3). Means in one column with diverse superscripts show significant differences (*p* < 0.05). Abbreviations: ΣHUFA, total polyunsaturated fatty acids; ΣMUFA, total monounsaturated fatty acids; ΣSFA, total saturated fatty acid.

## Data Availability

The data are available from the corresponding author upon reasonable request.
